# A Multicenter Study of Beta-Lactamase Resistant *Escherichia coli* and *Klebsiella pneumoniae* Reveals High Level Chromosome Mediated Extended Spectrum **β** Lactamase Resistance in Ogun State, Nigeria

**DOI:** 10.1155/2014/819896

**Published:** 2014-03-24

**Authors:** Folasoge A. Adeyankinnu, Babatunde O. Motayo, Akinniyi Akinduti, John Akinbo, Joseph I. Ogiogwa, Bukola W. Aboderin, R. A. Agunlejika

**Affiliations:** ^1^Microbiology Unit, Pathology Department, Federal Medical Centre, Abeokuta, Nigeria; ^2^Department of Vet Microbiology and Parasitology, Federal University of Agriculture, Abeokuta, Nigeria; ^3^Department of Medical Microbiology and Parasitology, Obafemi Awolowo University Teaching Hospital, Ile-Ife, Nigeria

## Abstract

As a result of the ever increasing problem of multiresistant bacteria, we instituted a surveillance program with the aim of identifying the basic molecular properties of ESBL in our environment. About 197 isolates of *Escherichia coli* and *Klebsiella pneumoniae* were selected and tested for ESBL production and antimicrobial susceptibility. Plasmid profiles were determined and curing ability was tested. ESBL prevalence was 26.4% for all isolates tested, with *E. coli* having a greater proportion. There was absolute resistance to ampicilin, tetracycline, and co-trimaxole among tested isolates. There was above average susceptibility to the 2nd and 3rd generation cephalosporins. Plasmid profiles of tested isolates ranged from 9 kbp to 26 kbp with average of 14.99 ± 2.3 kbp for *E. coli* and 20.98 ± 1.8 kbp *K. pneumoniae*, 9.6% of ESBL positive *E. coli* plasmids were cured, while 3.9% of *K. pneumoniae* plasmids were cured after treatment. The present study shows an upsurge in ESBL acquisition by gram negative bacteria and evidence of cocirculation of varying subtypes of ESBL with both plasmid transmissible and chromosome encoded subtypes. This calls for universal surveillance and more effort towards molecular epidemiology of this public health treatment.

## 1. Introduction

Extended spectrum *β*-lactamase arises as a result of mutations in certain genes such as TEM, SHV, CTX-M, and other genes that are commonly found in the Enterobacteriaceae family [1]. The mutation causes an alteration of their amino acid configuration thus conferring on these enzymes the ability to hydrolyze a wider spectrum for beta-lactam antibiotics including penicillin, oxyiminocephalosporins such as cefotaxime, ceftazidime, and ceftriaxone, and monobactam, for example, aztreonam [2]. Enterobacteriaceae are ubiquitous organisms that are found worldwide in soil, water, and vegetation and are part of the normal intestinal flora of most animals including human. These bacteria cause a variety of human diseases, including 30% to 35% of all septicemias, more than 70% of urinary tract infections, and many intestinal infections [3].

Majority of ESBL-producing organisms are* Escherichia coli* and* Klebsiella pneumoniae*. Others include* Enterobacter *spp.,* Salmonella *spp.,* Morganella*,* Proteus mirabilis*,* Serratia marcescens*, and* Pseudomonas aeruginosa* [4]. ESBL-producing strains often exhibit a multidrug resistant phenotype, including resistance to aminoglycosides and fluoroquinolones, further limiting the therapeutic options available to the clinicians [5].

ESBL-producing strains have been isolated from abscesses, blood, catheter tips, lungs, and peritoneal fluid, sputum, and throat cultures [6]. They apparently have a worldwide distribution. Rate of isolation varies greatly worldwide and within geographic areas and is rapidly changing over time [6].

Infections with these ESBL-producing organisms continue to be associated with limited therapeutic options, resulting in higher mortality and morbidity rate as well as high cost of treatment. In a recent study by Olowe and Aboderin [7], ESBL was detected in about 8% of tested isolated at a major hospital in Abeokuta, hence resulting in the need to further study the general ESBL prevalence rate in Abeokuta metropolis and determine their basic molecular properties. It is with this need that the antimicrobial surveillance system was set up at the Federal Medical Centre Abeokuta. This is the first surveillance report, evaluating the prevalence of ESBL-producing organism isolated from major hospitals in Abeokuta and their basic molecular properties.

## 2. Materials and Methods

### 2.1. Study Area and Study Population

Abeokuta Township was the study area chosen. It was the capital of Ogun State which is located at rain forest belt of latitude 43°E and longitude 37°S of the South Western Nigeria with population of about 3.5 million people [8].

The study was carried out on clinical isolates obtained from Microbiology Units of Federal Medical Centre, Idi-Aba, Ogun State General Hospital, Ijaiye, and Sacred Heart Hospital Lantoro, Abeokuta, Ogun State. Federal Medical Centre is the largest of the 3 study centres with a capacity of about 400 bed spaces; it also serves as a referral centre for Ogun State General Hospital, Ijaiye, and Sacred Heart Hospital Lantoro. The study duration was from November 2012 to May 2013.

### 2.2. Sample Collection and Sample Size Determination

The isolates were from clinical samples such as urine, wound swabs, aspirates, blood, high vaginal and endocervical swabs, and sputum obtained from various patients attending the hospital and their data was obtained. For the purpose of this study samples were selected based strictly on institutional bases; patients on referral from any other hospital were excluded, to prevent accidental overlap of isolates from the same patients visiting 2 different centres. The sample size was determined using the formula derived by [9]

(1) $$\begin{array}{c} \text{Sample}\;\text{Size}\;\left(n\right)=Z^{2}*\left(P\right)*\frac{\left(1-P\right)}{C^{2}}, \end{array}$$

where

(2) $$\begin{array}{c} Z=1.96\;\left(\text{for}\;95\%\;\text{confidence}\;\text{level}\right). \end{array}$$

*P* = Prevalence rate of 11.4% (0.114) in a similar study in UNTH, Enugu, to determine the prevalence rate of extended beta-lactamase producing gram negative bacilli (GNB) Enterobacteriaceae. Consider

(3) $$\begin{array}{c} c=\text{Confidence}\;\text{interval},0.05. \end{array}$$

### 2.3. Bacteria Isolation and Identification

All the samples collected were cultured within 2 hours of collection on Blood agar and Mac Conkay agar (Oxoid CM 516, UK) and Drigalski Lactose agar with ceftazidime and incubated at 37°C for 18–24 hours aerobically. Each organism was identified according to Cowan and steel method of bacteria identification [10].

### 2.4. Antimicrobial Susceptibility Testing

Antimicrobial susceptibility was determined by Kirby-Bauer disk diffusion method as per CLSI recommendations [11]. Antibiotic discs used are ampicillin (10 *μ*g), amoxicillin clavulanic acid (20/10 *μ*g), ceftriaxone (30 *μ*g), ceftazidime (30 *μ*g), gentamycin (10 *μ*g), ofloxacin (25 *μ*g), and imipenem (30 *μ*g).

### 2.5. ESBL Detection

Isolates were tested for beta-lactamase production using acidometric method as described earlier [12]. All positive *β*-lactamase isolates were screened for ESBL production by double disk diffusion test according to CLSI criteria [11, 13]. Briefly isolates were streaked unto Mueller Hinton agar and ceftazidime disk (30 *μ*g) and cefotaxime (30 *μ*g) were placed alone and in combination with clavulanic acid (10 *μ*g) (Oxoid UK), 2 cm apart. A difference of ≥5 mm between the zones of inhibition of the ceftazidime disk alone and the ceftazidime disk in combination with clavulanic acid was taken to be ESBL positive.* Escherichia coli* ATCC 25922 and* Klebsiella pneumoniae* ATCC 70603 were used as controls.

### 2.6. Plasmid Profiling

Plasmid extraction was done as previously described [12, 14]. Extracted plasmid DNA was loaded unto 0.8% Agarose gel. The resulting gel electrophoresis was visualized in a UV Trans-illuminator and molecular weight distances were determined according to Kim and Lim [15] (see Figure 2).

### 2.7. Plasmid Curing

**Figure 1:**
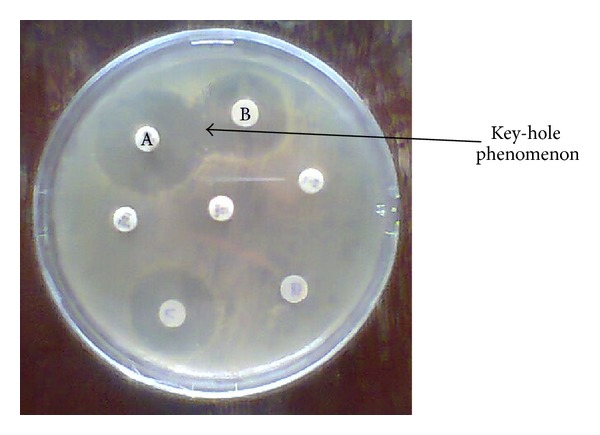
Culture plate showing synergy of clavulanic acid containing disk with ceftazidime in the double disk synergy test (DDST) for ESBL detection. A is augmentin + ceftazidime. B is ceftazidime alone. Zone of inhibition of A − zone of inhibition of B ≤ 5 mm; it is ESBL negative. Zone of inhibition of A − zone of inhibition of B ≥ 5 mm; it is ESBL positive.

**Figure 2:**
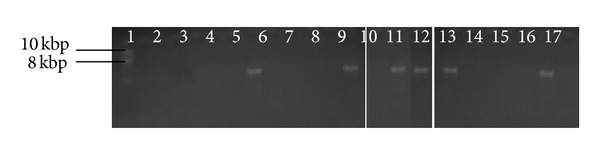
Agarose gel electrophoresis of plasmid DNA after curing.

ESBL positive isolates were selected and subjected to acridine orange (Merck) plasmid elimination as previously described [16]. Briefly each tested organism was grown in 5 mL double strength Mueller Hinton broth supplemented with 0.1 ng mL^−1^ acridine orange and incubated at 37°C for 24 hrs. After incubation test organisms were retested for ESBL production using DDST (see Figure 1).

## 3. Results

A total of 197 bacteria isolates (*Escherichia coli* and* Klebsiella pneumoniae*) were collected from different clinical samples from three hospitals: Federal Medical Centre, State Hospital Ijaiye, and Sacred Heart Hospital in Abeokuta Ogun State, South Western Nigeria.

The frequency of bacterial isolates obtained from clinical samples is shown in Tables 1 and 2. A total of 12 different clinical samples were collected from the hospitals. Out of 197 isolates obtained, 135 (68.5%) were* Escherichia coli* and 62 (31.5%) were* Klebsiella pneumoniae*. The highest number of isolate 73 (37%) bacteria isolates were obtained from urine samples yielded the highest number of isolates of 73 (37%) (Table 2),* Escherichia coli* having 61 (45.2%) and* Klebsiella pneumonia* 12 (19.4%).

Table 1 showed the frequency of isolates in relation to sites. The highest percentage of* Escherichia coli* isolates (49.7%) was obtained from Federal Medical Centre, Idi-Aba, while (37.0%) and (13.3%) were obtained from State hospital Ijaye and Sacred heart hospital Lantoro respectively. Total of 30 (48.4%) of the* Klebsiella pneumoniae* isolated were obtained from Federal Medical Centre, Idi-Aba, and 22 (35.5%) from State Hospital Ijaiye while 10 (16.1%) were obtained from Sacred Heart Hospital Lantoro.

Table 2 shows frequency rate of ESBL positive* E. coli* and* Klebsiella pneumoniae* in various clinical samples. The highest percentage (7.1%) of* E. coli* producing ESBL was found in urine samples while the lowest percentage of 1.0% was found in catheter tips, wound, pus, and pleural fluid, respectively; none was found in CSF, semen, HVS, and sputum. The prevalence rate of 18.3%* E. coli* producing ESBL was found in all the samples. The highest number of 2.5% of* Klebsiella pneumoniae* was found in urine samples followed by 2.0% in sputum samples and lowest rate of 1.0% in blood and pus samples while no* Klebsiella pneumoniae* producing ESBL was found in CSF, semen, ECS, HVS, and catheter tip. The prevalence rate of 8.1% ESBL positive* Klebsiella pneumoniae* was found in all the samples. Rate of occurrence of ESBL production was tested for statistical significance, 52 (26.4%) of the isolates were ESBL positive while 145 (73.6%) of the isolates were non ESBL, *T* = 0.09, *P* = 0.03, with a statistical significant between ESBL producers and non ESBL producers.

Result of antibiotic susceptibility: antibiotic susceptibility rate of ESBL isolates obtained from different clinical samples to commonly used antibiotics by disc diffusion test. Among the ESBL positive* E. coli* and* K. pneumoniae* isolates were 100% resistant to ampicillin, cotrimoxazole, and tetracycline while being 25% resistant was noted to nitrofurantoin and 50% to ofloxacin. There is 100% susceptibility to imipenem by both ESBL-producing isolates.

Result of plasmid analysis: Table 3 shows the result of Antibiotic susceptibility test of ESBL isolates obtained from different clinical samples to commonly used antibiotics by disc diffusion test. Among the clinical samples showed that highest average weight of 24.3 kbp and 22.1 kbp was in* E. coli* and* K. pneumoniae* producing ESBL from catheter tips and sputum samples, respectively. Low average weight of 10.3 kbp and 19.9 kbp was found in* E. coli* and* K. pneumoniae* producing ESBL from pus and blood samples, respectively, as shown in Table 4. No plasmid was found in some other* E. coli* and* K. pneumoniae* producing ESBL isolates. The average resistant plasmid weight of all the* E. coli* was 14.99 kbp ± 2.3 and* K. pneumoniae* was 20.98 kbp ± 1.8.

Table 5 shows the plasmid curing rate for the ESBL positive* E. coli* and *K*.* pneumonia*. Out of 36 ESBL-producing* E. coli* isolates, only 5 (9.6%) were cured and only 2 (3.9%) out of 16 ESBL-producing* K. pneumoniae* were also cured; that is, their plasmids were removed.

## 4. Discussion

Though ESBL might be produced by several members of the family Enterobacteriaceae, the present study was restricted only to detect their presence in clinically significant* Escherichia coli* and* Klebsiella pneumonia*e isolates. One hundred and ninety-seven bacterial isolates were selected. One hundred and thirty-five (68.5%) were* Escherichia coli* and sixty-two (31.5%) were* Klebsiella pneumoniae.* The highest number of bacterial isolates was obtained from urine samples, out of which sixty-one (45.2%) were* Escherichia coli* while twelve (19.4%) were* Klebsiella pneumoniae*. Urine had the highest number of both* Escherichia coli* and* Klebsiella pneumoniae* in the study which is in agreement with a study by Iroha et al. [16].* Klebsiella pneumoniae* is also recognized as an etiological agent of pneumonia, urinary tract infection, and nosocomial infections [17].* Escherichia coli* which is one of the most common causes of urinary tract infection and other opportunistic infections such as wound abscess which can have serious clinical implication [18, 19] had the highest percentage in this study.

The highest number of bacterial isolates was recovered from Federal Medical Centre followed by State Hospital Ijaiye and Sacred Heart Hospital Lantoro, respectively. Federal Medical Centre is a tertiary medical institution in Abeokuta, where patient inflow is high and more critical cases are being referred, while the state hospital and Sacred Heart Hospital are secondary medical institutions in which critical cases are not as high. Generally from the study the result in Table 3 shows that 75 (55.6%) of the* Escherichia coli* isolates were obtained from male clinical samples and 60 (44.4%) from female ones; likewise 28 (45.2%) and 34 (54.8%)* Klebsiella pneumoniae* was obtained from both genders. There is no evidence to show whether there is significance in the percentage or number of isolates collected from both genders since isolates collected were not gender based for ESBL production. Age group ≤10 years had the highest percentage of* Escherichia coli,* 38 (28.1%), and* Klebsiella pneumoniae,* 12 (19.4%), compared to other age groups, which was in accordance with a previous study done by Olowe and Aboderin [7].

In this study, the prevalence rate of ESBL-producing isolates of* Escherichia coli* was 18.3% and of* Klebsiella pneumoniae* was 8.1% giving the overall prevalence of 26.4%. The recorded prevalence rate of 26.4% is higher than 11.4% obtained in Enugu, Enugu State, South East Nigeria, by Iroha et al. [16] and similar to 25% rate obtained in Lagos, Nigeria, by Aibinu et al. [20]. This is also higher than 7.5% prevalence rate obtained by Olowe and Aboderin [7] in South West Nigeria. Similarly, a very high rate has been reported in developed countries such as 40% in The Netherlands [21], 51% in China [22], and 86.6% in India [23].

The highest occurrence of* Escherichia coli* and* Klebsiella pneumoniae* producing ESBL in this study was from urine samples, 19 (9.6%), followed by blood, 8 (4.0%), while cerebrospinal fluid, semen, and high vaginal swab were negative for ESBL enzyme production. In the study carried out by Olowe and Aboderin [7], there was an occurrence of (8/9) of ESBL from urine and (1/9) from other samples. A much higher prevalence rate of ESBL producers from urinary isolates of gram negative bacilli (58%) was previously reported in India by Duttaroy and Mehta [24]. Past studies have indicated that most patients with ESBL positive strains had urinary tract infection (27.9%), followed by sepsis (17.9%) and other medical conditions [25], which is in accordance with our study. The relatively high ESBL occurrence among isolates from blood culture is worrisome. A case of a probable ESBL outbreak at the Pediatrics Department of Federal Medical Centre Abeokuta has been previously observed (Adediran 2011, unpublished data). A similar finding was also observed in our current study with majority of isolates showing CTX-M and TEM phenotype by DDST and susceptibility pattern; we found a high ESBL rate among blood culture isolates in pediatrics (*E. coli, n* = 4;* K. pneumoniae, n* = 2), out of a total of 8 positive isolates (Table 2). However, owing to lack of facilities carrying out Pulse Field Gel Electrophoresis (PFGE) analysis in our study setting, further molecular characterization of ESBL positive isolates could not be done. Isolates from clinical samples, blood, and urine have the highest percentage of ESBL positive which is 4.0% and 9.6%, respectively. In this study, majority of the ESBL positive blood culture isolates were recovered from children (Table 2), many of whom were exposed to infection as a result of poor infection control practices in our study setting as previously reported [12]. However 2 ESBL blood culture isolates were also recovered from adult hospitalized patients, whom had been catheterized. These adults most likely developed bacteraemia as a result of catheter associated urinary tract infection. All patients with suspected septicemia however recovered and were discharged after treatment with appropriate antibiotics. In the present study the ESBL isolates obtained from various samples were significant at *P* = 0.03 at 95% confidence interval using *X* ^2^ test.

It was observed from this study that imipenem had no resistance rate to both ESBL producing* Escherichia coli* and* Klebsiella pneumoniae*, 0% (*n* = 52) and non-ESBL strains too 0% (*n* = 142) which in consonance to previously reported research [26]. Both the ESBL- and the non-ESBL-producing strains of* Escherichia coli* and* Klebsiella pneumoniae* isolates were found to be resistant to three of the commonest antibiotics in use in our environment, namely, ampicillin, tetracycline, and cotrimoxazole. This calls for urgent action with regard to education of the public against the misuse of antibiotics and strict compliance to the antibiotic regimen. Most often, isolates possessing these enzymes also exhibit resistance to fluoroquinolones, aminoglycoside, sulphonamides, and tetracycline [27]. This also correlates with the study done by Denholm et al. [28] and Jabeen et al. [29]. This is because genes coding for beta-lactamases are often located on large plasmids that also encode genes for resistance to other antibiotics including aminoglycosides, tetracycline, and quinolones [12]. In this study there is such associated resistance with gentamicin (54% and 63%) for both* E. coli* and* K. pneumoniae,* respectively, and 100% resistance to tetracycline and cotrimoxazole for both isolates.

In our study, the R-plasmid profile of ESBL positive* Escherichia coli* and* Klebsiella pneumoniae* among the clinical samples was conducted and showed the average weight of 20.71 ± 2.3 kbp and 25.10 ± 1.8 kbp for* Escherichia coli* and* Klebsiella pneumoniae,* respectively which shows that majority of the ESBL producing strain harbored resistance plasmid with high level resistance to beta-lactam antimicrobial agents which pose a dangerous threat to effective therapy. A very heavy plasmid size was exhibited by all the isolates of* Escherichia coli* and* Klebsiella pneumoniae* with plasmid size of not less than 6.7 kbp and 13.2 kbp, respectively. These findings are in agreement with the report of a study by Sharma et al. [30], stating that ESBL producers express their beta-lactamase genes from plasmids that also encode resistance to other antibiotics such as aminoglycoside, sulphonamide, tetracycline, and other antibiotics.

A study by Kim and Lim [15] reported that since ESBL producers express their *β*-lactamase genes from plasmids, genes encoding for ESBL resistance and resistant phenotypes to other class of antibiotics may reside within the same plasmid and can therefore spread together. However the result obtained in our study showed that 7 (13.5%) plasmids out of 52 positive ESBL isolates were cured and 45 (86.5%) were not cured. It is clearly observed from our study that resistant property is borne not only within the plasmid but also within the chromosomes which is in accordance with a previous study done by Iroha et al. [16]. This also confirms the report made by Bradford [1] that many species of gram negative bacteria possess naturally occurring chromosomally mediated *β*-lactamase enzymes.

## 5. Conclusion

The present study reveals an upward surge in the prevalence of ESBL resistance in commonly encountered gram negative pathogens. This call for enforcement of policy guided antimicrobial regulations aimed at more responsible antibiotic prescriptions and regulated distribution as is done in the developed countries. Hospital infection control committees should also be strengthened particularly in our region. Molecular surveillance and epidemiology of this class of resistant bacteria are also advocated.

## Figures and Tables

**Table 1:** Frequency of isolates (*n* = 197) in relation to sites.

Study site	*Escherichia coli *		*Klebsiella pneumonia *	
	Number of isolates	Percentage (%)	Number of isolates	Percentage (%)
F.M.C	67	49.7	30	48.4
Ijaiye	50	37.0	22	35.5
Lantoro	18	13.3	10	16.1

Total	135	100	62	100

**Table 2:** Frequency rate of ESBL positive *E. coli* and *Klebsiella pneumoniae* in various clinical samples.

Samples	Number of isolates	ESBL positive isolates			
		*Escherichia coli *		*Klebsiella pneumoniae *	
		Number (*N*)	Percentage (%)	Number (*N*)	Percentage (%)
Blood	26 (*n* = 24)*	6	3.0	2	1.0
Urine	73	14	7.1	5	2.5
CSF	9 (*n* = 9)*	0	0	0	0
Semen	3	0	0	0	0
Endocervical	9	4	2.0	0	0
HVS	4	0	0	0	0
Catheter	9	2	1.0	0	0
Sputum	12	0	0	4	2.0
Ear swab	12 (*n* = 5)*	4	2.0	0	0
Wound	20	2	1.0	0	0
Pus	8	2	1.0	2	1.0
Pleural fluid	12	2	1.0	3	1.5

Total	197 (*n* = 38)*	36	18.3	16	8.1

NB: *Isolates recovered from pediatric subjects.

**Table 3:** Antibiotic susceptibility rate of ESBL isolates obtained from different clinical samples to commonly used antibiotics by disc diffusion test.

Antibiotic	*Escherichia coli* (*n* = 36)							*Klebsiella pneumonia* (*n* = 16)					
	(ug/disc)	*S*	%	*I*	%	*R*	%	*S*	%	*I*	%	*R*	%
AMP	10	0	0	0	0	36	100	0	0	0	0	16	100
OFLX	5	16	44.4	2	5.6	18	50	6	37.5	4	25	06	37.5
AUG	20/10	9	25	2	5.6	25	69	4	25	2	12.5	10	63
COT	5/25	0	0	0	0	36	100	0	0	0	0	16	100
GEN	5	13	36.1	4	11.1	19	54	5	31.2	1	6.3	10	63
NIT	30	20	55.6	5	13.9	09	25	8	50	2	12.5	06	37.5
TET	30	0	0	0	0	36	100	0	0	0	0	16	100
CAZ	10	3	8.3	3	8.3	30	83	4	25	2	12.5	10	63
CRO	10	5	13.9	3	8.3	28	78	5	31.2	3	18.8	08	50
CXM	10	1	2.8	3	8.3	32	89	0	0	4	25	12	75
IMP	5	36	100	0	0	0	0	16	100	0	0	0	0

**Table 4:** R-plasmid profile of ESBL positive *Escherichia coli* and *pneumonia* among the clinical samples.

Clinical samples	Average plasmid size (kbp)	
	*E. coli *	*K. pneumonia *
Blood	14.6	19.9
Urine	15.8	21.7
CSF	0	0
Semen	0	0
ECS	13.8	0
HVS	0	0
Catheter	24.3	0
Sputum	0	22.1
Ear	13.0	0
Wound	17.0	0
Pus	10.3	21.2
Pleural fluid	11.1	20.0
Average weight	20.71 ± 2.3	25.10 ± 1.8

**Table 5:** Plasmid curing rate for the ESBL positive *E. coli* and *K*. *pneumoniae*.

	Number of isolates with plasmid	Number of isolates with no plasmid after curing	Percentage of isolates cured (%)
*E. coli *	36	5	9.6
*K. pneumonia *	16	2	3.9

Total	52	7	13.5
